# Application and Effects of Ohmic-Vacuum Combination Heating on the Quality Factors of Tomato Paste

**DOI:** 10.3390/foods10122920

**Published:** 2021-11-25

**Authors:** Zina T. Alkanan, Ammar B. Altemimi, Asaad R. S. Al-Hilphy, Francesco Cacciola, Salam A. Ibrahim

**Affiliations:** 1 Department of Food Science, College of Agriculture, University of Basrah, Basrah 61004, Iraq; zina.alkanan@uobasrah.edu.iq (Z.T.A.); aalhilphy@yahoo.co.uk (A.R.S.A.-H.); 2 Department of Biomedical, Dental, Morphological and Functional Imaging Sciences, University of Messina, Via Consolare Valeria, 98125 Messina, Italy; cacciolaf@unime.it; 3 Food and Nutritional Science Program, North Carolina A & T State University, Greensboro, NC 27411, USA; ibrah001@ncat.edu

**Keywords:** tomato, phytochemicals, ohmic-vacuum combination, microbiological stability

## Abstract

Ohmic-vacuum combination heating is a common method used in the food industry as a concentration process. In the present study, an OH-VC combination heating system was developed for producing tomato paste at temperatures of 70, 80, and 90 °C and pressure of 0.3, 0.5, and 0.7 bar and electric field of 1.82, 2.73, and 3.64 V/cm using a central composite design. The effects of heating conditions on the quality and sensory evaluation of tomato paste were also evaluated. Each combination of temperature, pressure, and the electric field was quantified for specific energy consumption, energy efficiency, and productivity. A decrease of 35.08% in the amount of acid ascorbic and lycopene content 19.01%, using conventional heating compared to ohmic-vacuum heating under optimized conditions, was attained. The results also highlighted an increase in the amount of HMF (69.79%) and PME (24.33%) using conventional heating compared to ohmic-vacuum heating under optimized conditions. Ascorbic acid, lycopene, titratable acidity, productivity, energy efficiency was higher than conventional heating; on the other hand, HMF, PME, pH, SEC were lower than conventional heating at the applied OH-VC process. No significant effects between OH-VC and conventional heating on the TSS were observed. In addition, OH-VC heating was highly efficient in the inhibition of bacterial growth. Further, a minor effect on the sensory properties of tomato paste with OH-VC heating compared to the conventional treatment. The obtained results indicate a strong potential for an OH-VC combination heating system as a rapid-heating, high-efficiency alternative for saving electrical energy consumption and preserving nutritional value.

## 1. Introduction

Tomato (*Lycopersicon esculentum Mill*) is one of the most widely consumed cultivated horticultural crops globally and ranks second in global production among vegetable crops [1]. Tomato cultivation is prominent in the governorates of Basrah, Karbala, Nineveh, Baghdad, and Najaf; tomatoes grow either in temperate and hot regions or in greenhouses that maintain optimal growing temperatures [2]. Moreover, in Iraq alone, tomato production was recently estimated at 468 thousand tons with a ratio of 33.9% of the total production of crops and other vegetables from a cultivated area of 6709.8 k/ton. The total production of all governorates was estimated as 467,579 tons [3].

Tomato undergoes a variety of different treatments aiming to transform it into well-known commercial products such as sauce, ketchup and juice, canned whole tomatoes, and tomato paste [4,5]. For example, for preservation after harvesting, tomatoes are thermally treated with several techniques [6], such as pasteurization and sterilization [7], and drying and evaporation at high temperatures, which help to ensure the microbiological safety and stability of the resulting products; however these methods reduce the nutritional value thus negatively affecting the product quality [8]. Recent studies were carried out to enhance the quality, safety, and shelf life of tomatoes following electrical technologies e.g radio frequency, sound waves, high-pressure thermal sterilization, and infrared heating, hydrostatic high-pressure dielectric heating, ohmic heating, irradiation, pulsed electric field, and high voltage, ultrasound, cold plasma, c, inductive forced heating and pulsed electric field [9,10].

Conventional heat treatments are currently the most common ones in the food industry, but due to the heat transfer mechanism (conduction and convection), these processes have some drawbacks such as high temperature, loss of nutritional value, and sensory changes. In addition, the combustion of the fuel used to generate heat results in economic energy losses. These disadvantages can be avoided with modern technologies such as ohmic heating [11]. The latter is a thermal treatment that heats food by passing an alternating electric current through it; as a consequence, this method has a large capacity for rapid and homogeneous heating [12]. In addition, this technique provides safe and high-quality food products [13] while preserving their nutritional value [10]. Moreover, some studies have shown that ohmic heating enhances food safety and extends the shelf life without the addition of additives with a minor impact on the organoleptic properties [14]. Ohmic heating technology is characterized by high energy allowing to reduce heating time by 90–95% [15]. Others studies have demonstrated great potential for using ohmic heating as an alternative to traditional heating methods for many food processing applications [16].

Vacuum evaporation is another common method used in the food industry as a concentration process. This technique can be carried out at temperatures with negative effects on food products, compared to atmospheric evaporation even though they require a longer processing time and more energy consumption [17]. As a consequence, vacuum evaporation should be more efficient in order to increase the effectiveness of its use in evaporation systems under vacuum and to reduce the loss of nutritional value [18].

Many applications can be found in the literature for ohmic treatments e.g., developing probiotic dairy foods, extracting bioactive phenolic compounds, infant formula processing, water distillation, pasteurization of strawberry flavored whey syrup, thawing frozen tuna, inhibition of microorganism growth, fruit and vegetable processing, and evaporation, dehydration, and cooking [19]. Considering the rapid deterioration of tomatoes after harvesting several thermal treatment techniques have been used in order to prolong preservation and storage periods for conversion into other safe, stable, and more popular products [20]. During its concentration stages, tomato treatment is affected by sterilization, which is responsible for the color changes and ascorbic acid deterioration [21]. Consequently, the quality of producing tomato paste depends on the effect of the temperature used on the soluble solids, sugar, acidity, color, and pH [6].

The present study was conducted in order to develop an OH-VC combination heating system for producing tomato paste for farms in southern Iraq. In addition, the investigation of the effect of an OH-VC combination heating system compared to the conventional heating on ascorbic acid content, lycopene, hydroxymethylfurfural, pectin methylesterase, pH titratable acidity, total soluble solids, and microorganism inactivation was investigated.

## 2. Materials and Methods

### 2.1. Sample Preparation and Processing

**Figure 1:**
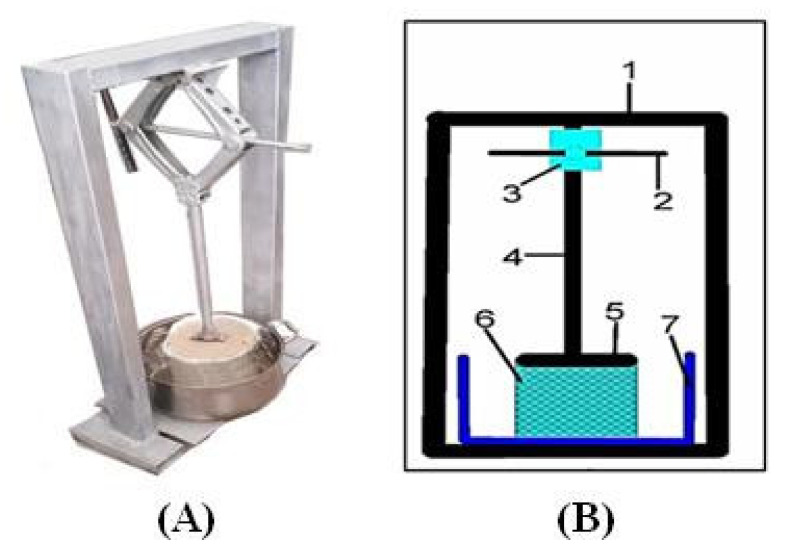
(**A**): Laboratory-made mechanical piston, (**B**): Engineering drawing of a mechanical piston, 1. Framework; 2. Lever; 3. Mechanical press; 4. Steel shaft; 5. Pressure disc; 6. Perforated cylinder; 7. Juice collecting tank.

A 5 kg ripe tomato was selected, washed, and subjected to a cleaning process in order to eliminate dust and dirt and to reduce the microbial load. A cold crushing process was used, and the tomato was placed in a manual device (the mechanical compressor) (Figure 1) after cutting in order to isolate the juice from the peels and seeds. The filtration process was carried out using sterilized gauze to remove impurities, particles, seeds, and husks. After the filtering process, the weighed tomato juice was approximately 4.660–4.800 kg, while the seeds and peels were 0.200–0.340 kg. The tomato juice was then heated using a manufactured ohmic-vacuum (OH-VC) combination device. The control panel was used to set the temperature, pressure, and electrical field for 20 treatments (70, 80, 90 °C) and (0.7, 0.5, 0.3 bar) and (1.82, 2.73 and 3.64 V/cm), respectively. The tomato paste was transferred into a clean, sterilized glass container and kept at 4 °C until needed.

### 2.2. Conventional Heating

The conventional heating treatment was carried out using the double jacket vat through a heat transfer fluid (water) in order to prevent direct contact with the heat source and to control the temperature that ranged from (87–90 °C).

### 2.3. Ohmic-Vacuum (OH-VC) Combination Heating Device

**Figure 2:**
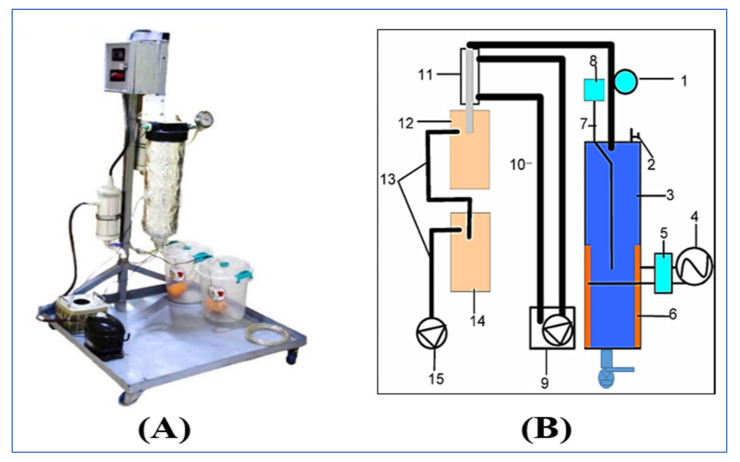
(**A**). Photograph of the actual ohmic-vacuum (OH-VC) combination heating device. (**B**). Schematic diagram of (OH-VC) consisting of 1. Pressure vacuum gauge; 2. Pressure vacuum calibration valve; 3. Heating cylinder; 4. Electricity source; 5. Variac; 6. Stainless steel electrodes; 7. Thermocouple; 8. Temperature controller; 9. Coldwater circulation unit; 10. Pipes; 11. Glass heat Exchanger; 12. Moisture trap; 13. Piping; 14. Moisture trap; 15. Vacuum pump; 16. Valve.

OH-VC combination heating device (Figure 2) was designed and constructed at the Food Engineering laboratory, Department of Food Science, College of Agriculture, University of Basrah, Basrah, Iraq. The device consisted of an ohmic heating cylinder, which was provided with a control valve, thermocouple, vacuum pressure gauge, and two rectangular electrodes. The other parts of the system were a vacuum pump connected with an ohmic heating cylinder via a plastic pipe, two hunters which were used to separate water from the air, a glass condenser used to condense vapor to liquid (water), a control panel, and an in-system cleaning device. The cylindrical ohmic heating chamber (thickness of 1.5 cm, diameter of 27 cm, height of 85 cm, and capacity of 8 L) was made of heat-resistant plastic. Stainless steel type 316 (dimensions of 35 × 10 cm) rectangular electrodes were fixed into the inner wall of the cylindrical ohmic chamber. The heat generation system generated alternating current (AC) and voltage ranging from (0–220 V) and (50 Hz) frequency (50 Hz) which was controlled by a voltage regulator. The operation pressure in the ohmic heating cylinder ranged between 0.3–0.7 bar (lower than the atmosphere pressure). The tomato paste was collected in a stainless steel 10 L tank.

### 2.4. Physicochemical Analysis

#### 2.4.1. pH Determination

5 g of tomato paste was blended with 10 mL of deionized water. The mixture was placed in an ultra-homogenizer and heated to 100 °C. The pH was determined after the mixture cooled to 20 °C [22].

#### 2.4.2. Titratable Acidity

Tomato paste (10 g) was mixed with 10 mL of distilled water and heated to 100 °C in order to eliminate CO_2_ [23]. The solution was then titrated with 0.1 N NaOH to pH 8.03 [6], and acidity was estimated as a percentage of citric acid by the following Equation (1):

Titrable acidity (% citric acid) = (V_NaOH_ × C_NaOH_ × 0.070 × 100)/V_sample_ (1)

where V_NaOH_ = titratable volume of solution; V_sample_ = titratable volume of sample; C_NaOH_ = concentration of NaOH solution.

#### 2.4.3. Total Soluble Solids (TSS)

Total soluble solids (TSS) were evaluated using a refractometer (Bellingham + Stanly, Bellingham, UK). A drop of the tomato sample was poured onto the refractometer prism (SCO, Dingetstädt, Germany).

#### 2.4.4. Density Determination

The density of the concentrated tomato juice was measured during the evaporation period (Equation (2)) as (kg/m^3^) using the values of the instantaneous temperature and the concentration of TSS. T represents the temperature in (K) [24].

(2) $$p_{t}=\left(0.79+0.35\times e^{0.0108\times \left(\mathrm{TSS}\right)}-5.41\times 10^{-4}\times T\right)\times 1000$$

### 2.5. Ascorbic Acid (AA) Content

For the determination of the AA content in the tomato paste, an iodine titration method [25,26] was adopted. Five g of tomato paste was weighed in a beaker, followed by the addition of 100 mL 2% hydrochloric acid. The mixture was left for 15 min prior to filtration (Whatman no. 1 paper). Five mL of this solution was filtered and 5 mL of distilled water and 3 mL of 1% potassium iodide were added. As an indicator, 2 mL of a fresh starch solution 1% (*w/v*) was employed. The mixture was then titrated with potassium iodate KIO_3_ (0.0017 M) until the tomato juice solution turned a clear, dark brown color. The determination of the AA content was carried out using the following Equation (3):

Ascorbic acid content (mg/100 mL sample) = 0.88 × iodine solution (mL)(3)

### 2.6. Lycopene Content

Crude lycopene extraction was performed and determined according to the method described by Ranganna [27] and Mai et al. [28]. Lycopene content was calculated using Equation (4).

Total Lycopene Content (μg/g) = A *×* volume (mL) *×* 104/A1%cm *×* sample weight (g) (4)

whereas A = absorbance; volume = total volume of extract, A1% 1 cm: absorption coefficient of lycopene in petroleum ether = 3450.

### 2.7. Hydroxymethyl Furfural (HMF)

For the determination of HMF, the following assay was performed as reported by Cohen et al. [29]. 95% ethyl alcohol (5 mL) was added to 5 mL of tomato juice sample, and the mixture was centrifuged at 1000× *g* for 15 min. The supernatant of the centrifuged sample was separated into two portions. For the HMF content, 2 mL of the other portion was poured into a 16 mL screwcap tube. Two mL of 12% *w*/*w* trichloroacetic acid (TCA; Sigma, Berlin, Germany) and 2 mL of 0.025 M thiobarbituric acid (TBA; BDH Limited, UK) were subsequently added. The tubes were placed in a water bath (Grant-UK) at 40 °C (±0.5 °C) and incubated for 50 min. Then, the tubes were cooled using tap water, and the absorbency was measured at 443 nm. A calibration curve of HMF (Aldrich, Germany) was used to quantify the HMF concentration.

### 2.8. Pectin Methyl Esterase (PME)

PME content was determined according to Kimball [30]. Equation (5) used for the PME was as follows:

(5) $$\mathrm{PME}\;(\mathrm{unit}/\mathrm{mL})=\frac{NaOH\;\left(0.05N\right)\;*\;0.1\;ml\;NaOH\;\left(0.05N\right)}{10\;ml\;of\;sample*time\;\left(minute\right)}$$

### 2.9. Electrical Conductivity

The electrical conductivity was calculated by recording the current by an ammeter during heating. The evaporation periods and electrical conductivity were monitored, and values were calculated using the following Equations (6) and (7) [31] icier 2003:

(6) $$EC=\frac{L\;I}{A\;V\;}$$

(7) $$A=\frac{m_{t}}{p\;L}$$

where *A* = the contact area (m^2^), $m_{t}=$ the mass of the sample (kg), p= the tomato density (kg/m^3^), *L* = the distance between the electrodes (m^2^), *I* = an electric current, *V* = voltage

### 2.10. Energy Efficiency Energy (%)

The energy efficiency used for the evaluation of the OH-VAC tomato paste concentrator was calculated according to the following Equation (8) [32]:

(8) $$\eta =100\times \left(\frac{m_{0\;}C_{p}\;\Delta T+m_{w}\times \lambda }{E}\right)$$

where η: energy efficiency (%), $C_{p:\;}$: the specific heat capacity (J/kg K), ΔT: the difference in temperatures (°C), $m_{w}:\;$ the mass of evaporated water (kg), λ:  the latent heat of vaporization of water (J/kg), E: energy consumption (J) (Magerramov, 2007).

The $C_{p}$ was calculated according to the following Equation (9) [31,32]:

(9) $$C_{p}=837+3349\;X_{w}$$

$X_{w}:$ Fraction of water.

### 2.11. Specific Energy Consumption

The specific energy consumption (SEC) was calculated in (J/kg water) according to the following Equation (10) [32]:

(10) $$SEC=\frac{\sum V\;I\;\Delta t}{m_{W}}$$

$m_{w}:$ the mass of evaporated water (kg); Δt: the difference in times (s).

### 2.12. Microbiological Analysis

Microbiological analysis was conducted using a serial dilution (10^−1^ to 10^−3^) method in which a sterilized glass tube was filled with 1 g of tomato paste, coliform bacteria, yeast, mold, and 9 mL of peptone water. 1 mL from each test tube was then poured onto the agar plates. The latter were incubated at 35 °C for 24–28 h and then subjected to TPC. For the estimation of coliform bacteria, a MacConkey agar was used, whereas in the case of yeast and mold potato dextrose agar was used with an incubation period of 3 days at 25–28 °C. The attained results were expressed based on log cfu/mL of tomato [33].

### 2.13. Sensory Analysis

Sensory evaluation of tomato paste quality was carried out in a Food Science laboratory according to international standard ISO 8589 [34,35]. The sensory evaluation was carried out by a panel of 10 trained judges in the Department of Food Sciences/College of Agriculture/University of Basrah under normal lighting conditions and room temperature 25 °C. Judges were asked to assess the color, texture, taste, appearance, and overall acceptability of tomato paste by applying a 9-point hedonic scale (1 = highly disliked to 9 = highly liked).

### 2.14. Experiment Design and Statistical Methods

Ohmic-Vacuum (OH-VC) combination heating device parameters were first optimized by adopting a response surface methodology (RSM) based on the central composite design (CCD). Temperature, pressure, and electrical conductivity with three levels (−1, 0, +1) were investigated. Specifically, temperatures were tested at 70, 80, 90 °C, pressures at 0.3, 0.5, 0.7 bar, and electrical conductivity at 1.82, 2.73, and 3.64 V/cm, respectively. In order to describe the effect of parameters using Design-Expert ver. 12 software, the following second-order polynomial model Equation (11) was employed:

(11) $$Y=\beta_{{}^{\circ}}+\overset{3}{\underset{i=1}{\sum }}\beta_{i}{\;X}_{i}+\overset{3}{\underset{i=1}{\sum }}\beta_{\mathrm{ii}}{\;X}_{i}{}^{2}+\sum \;\;\overset{3}{\underset{i<j=1}{\sum }}\beta_{\mathrm{ij}}{\;X}_{i\;}X_{j}$$

where Y is the response, X_1_ is the temperature, X_2_ is pressure and X_3_ is the electrical conductivity. For statistical analysis, the variance was determined (ANOVA; α = 0.05) in order to determine any differences among the optimized factors and conventional resistance heating. The coded values of the experiment factors and their settings for the experiment design are shown in Table 1, and the t data for all treatments can be seen in Table 2. All experiments were performed in triplicate, and data are presented as mean ± standard deviation (SD). Analyses were calculated using the Statistical Package of Social Sciences (SPSS) software package version 16.0. Data were assessed for significant differences employing one-way ANOVA and were statistically significant at *p* < 0.05.

## 3. Results and Discussion

### 3.1. Influence of OH-VC on pH and Titratable Acidity

Table 3 shows the pH values of treated tomato paste samples by ohmic heating using different conditions of the electric field (1.82–3.64 V/cm), temperatures (70–90 °C), and pressure (0.7–0.3 bar). The results highlight a few differences among all treatments for pH values (4.1–4.4). The highest pH value was at the highest values for the electric field, temperature, and pressure. This agreed with Darvishi et al. [36] who treated pomegranate juice using ohmic heating and found little changes in pH when using different values of the electric field. Munhoz and Schmidt [4] also reported that the pH values of tomato paste were (4.31 ± 0.01) using temperatures ranging from (60–80 °C). The statistical analysis revealed that the proposed model was adequate (R^2^ = 0.98) and lack of fit was not significant for the pH value (Table 4).

Table 5 shows the pH values of tomato paste using ohmic heating at the optimal condition (3.64 V/cm, 87.30 °C, 0.3 bar) for the electric field, temperature and pressure respectively. The results showed that there were no significant differences between ohmic heating and conventional heating with regard to the pH values. The results agreed with Monti [37], who indicated that pH 4.4 was the desired higher value when conducting heat treatments, and pH at 4.25 was the optimum value for tomato paste. The results also agreed with the findings of Darvishi et al. [38] who determined the pH of grape juice treated by ohmic heating which gave a lower pH value than grape juice treated with the conventional method.

Table 3 shows the percentage of titratable acidity in tomato paste treated with ohmic heating (between 0.32% and 0.46%). The highest measured titratable acidity (0.46%) was at 2.73 V/cm of the electric field, 90 °C, and 0.5 bar of pressure. This result was similar to those of [39] who estimated the titratable acidity range between 0.31% and 0.43% in tomato paste treated by ohmic heating and concluded that ohmic heating reduced acidity loss. The regression models for titratable acidity were significant with no significant lack of fit, indicating that the model was adequate for predicting both values.

**Figure 3:**
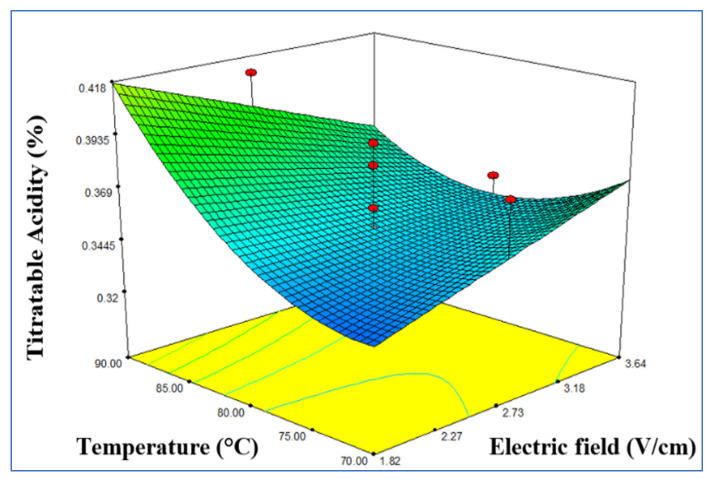
Response surface model plot showing the effects of independent variables on titratable acidity (%).

The interaction between the electric field and the temperature was significant on the titratable acidity percentage (*p* < 0.05), and statistical analysis revealed that the proposed model was adequate (R^2^ = 0.666). The titratable acidity increased when the temperature increased in the range of 70–90 °C, the pressure increased in the range of 0.3–0.7 bar, and the electrical field increased in the range of 1.82–3.64 V/cm (Figure 3).

Table 5 shows the titratable acidity values of tomato paste produced under the optimal conditions of ohmic heating at 3.64 V/cm of the electric field, 87.30 °C, and 0.3 bar. The titratable acidity of tomato paste under the optimum conditions of ohmic heating was 0.42% ± 0.0424 compared to the conventional heating 0.35% ± 0.0141%. The statistical analysis results revealed that there were significant differences between the ohmic heating and conventional heating regarding the obtained titratable acidity. It was noted during the study that the titratable acidity of tomato juice decreases with the increase in the treatment time and the temperatures used. This result was in agreement with Astuti et al. [40].

### 3.2. Effect OH-VC on TSS and Density

Table 3 shows the total soluble solids of tomato paste produced by ohmic heating, whose values ranged from 26 to 29% with different electric fields (1.82, 2.73, 3.64 V/cm), temperatures (70, 80, 90 °C), and pressures (0.7, 0.5, 0.3 bar). The content of total soluble solids was in accordance with the FAO/WHO FOOD standards program codex [41]. The results of the statistical analysis of the model were significant (*p* < 0.05) with no significant lack of fit (*p* > 0.05), which means that the linear model is suitable for predicting TSS, and the value of the CV was 2.48%, while the value of R^2^ was 0.90 (Table 4).

**Figure 4:**
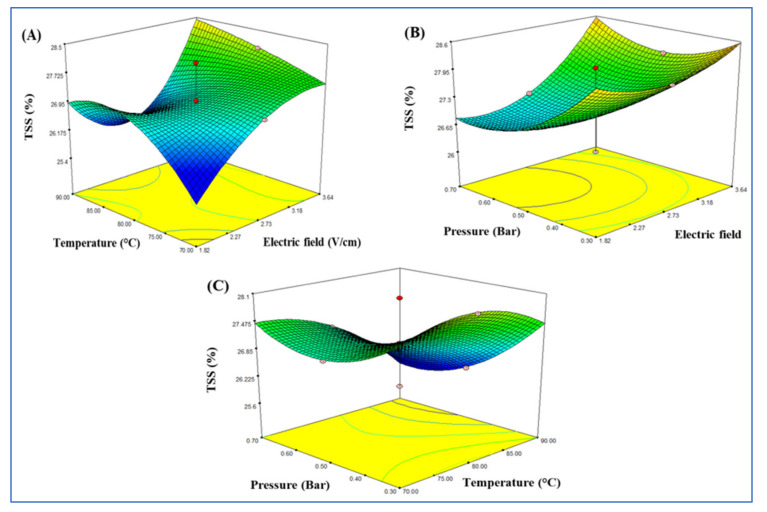
Response surface model plot showing the effects of independent variables on TSS (brix): panel (**A**), temperature and electric field; panel (**B**), pressure and electric field; and panel (**C**) temperature and pressure.

Figure 4 shows the response surface for TSS. The interaction between electric field and temperature, electric field strength and pressure, pressure and temperature had no significant effects on TSS. Poojitha and Athmaselvi [42] found a difference in TSS of papaya pulp treated with ohmic heating at different voltage gradients. This result was attributed to the water loss due to ohmic heating. Saberian et al. [43] also indicated that the amount of TSS was increased when the electric field was increased. Table 5 presents the TSS for produced tomato paste under the optimal conditions of ohmic heating at 3.64 V/cm of the electric field, 87.30 °C, and 0.3 bar compared with the conventional heating (87–90 °C). The results showed that there were no significant differences (*p* < 0.05) between ohmic heating and conventional heating. Each treatment needs a different period of time to reach the required concentration of TSS. The time frame for ohmic heating was only 95 min with the vacuum system, while the time period for conventional heating was much longer. These results were in agreement with Takeoka et al. [44] who indicated that tomato juice requires long treatment periods to reach the required concentration for TSS.

**Figure 5:**
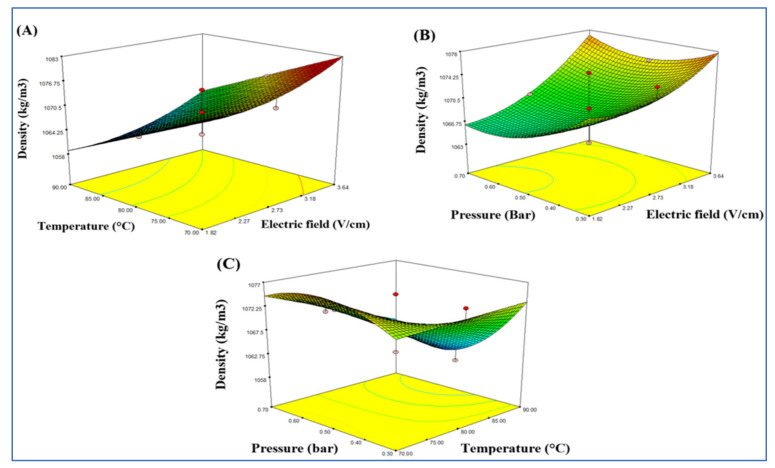
Response surface model plot showing the effects of independent variables on the density (kg/m^3^): panel (**A**), temperature and electric field; panel (**B**), pressure and electric field; and panel, temperature and pressure (**C**).

Table 3 shows the effect of ohmic heating on the density of tomato paste using different ranges of the electric field, temperature, and pressure (1.82, 2.73, 3.64 V/cm), (70, 80, 90 °C) and (0.7, 0.5, 0.3 bar), respectively. The results also revealed that the density values of tomato paste increased significantly (*p* < 0.05) when the temperature and the electric field were increased; no significant effects (*p* > 0.05) were observed from pressure. The regression models for lycopene content were significant (*p* < 0.05) differently from the lack of fit which was not significant (*p* > 0.05), indicating that the model was adequate for predicting both values. The value of the coefficient of determination (R^2^) was 0.87 (Table 4). Figure 5 shows the three-dimensional diagram of the response surface for the electric field, temperature, and pressure on the density. The interaction between electric field and temperature did not significantly affect (*p* > 0.05) the density. However, the interaction between temperature and pressure had a significant effect (*p* < 0.05) on the tomato paste density values.

### 3.3. Effect of OH-VC on Ascorbic Acid Content

Table 3 shows a clear difference in the amount of ascorbic acid in tomato paste produced from ohmic heating for each treatment based on the conditions used. The amount of ascorbic acid ranged (22–67.6 mg/100 mL) using different treatment combinations from the electric field, temperature, and pressure. The high temperatures and vacuum pressure at 90 °C and 0.7 bar, respectively, had a significant effect on the amount of ascorbic acid, while the reduction in the amount of ascorbic acid was small at temperatures (70 and 80 °C) and higher pressures (0.5, 0.3 bar). The high temperatures and vacuum pressure at 90 °C and 0.7 bar, respectively, had a significant effect on the amount of ascorbic acid, compared to lower temperatures (70 and 80 °C) and higher pressures (0.5, 0.3 bar). This finding was similar to that from previous studies [45,46]. The results were also in agreement with Hassen et al. [6] who found a decrease of (44%) in the amount of acid ascorbic compared to the two temperatures (70 and 80 °C). The decrease in the content of ascorbic acid was attributed to its ability to decompose when exposed to heat [47] gomathy. Changes in electric field values used in the treatments had no effect on the degradation of ascorbic acid, and this was in agreement with Assiry et al. [48].

Table 4 shows the analysis of variance for independent factors (electric field, temperature, and pressure) and the predicted ascorbic acid. The results showed that the model was significant (*p* < 0.05) as well as the value of lack of fit (*p* < 0.05), thus indicating that the electric field, temperature, and pressure had no significant effect on the ascorbic acid. Three-dimensional plots of surface responses (Figure S1) were generated by altering two variables within the experiment range while keeping the third variable constant at the central level. A significant increase in the amount of ascorbic acid was found using low temperatures, high-pressure, and low electric fields among all treatments.

Table 5 shows the comparison between the conventional treatment and ohmic heating under optimized conditions of the electric field (3.64 V/cm), temperature (87.30 °C), and pressure (0.3 Bar) with regard to the amount of ascorbic acid. The results showed that ascorbic acid deterioration under optimized conditions of ohmic heating was less than that with conventional heating. The ascorbic acid content was (67.76 ± 1.244 and 50.16 ± 1.244 mg/100 mL) under optimized conditions of the electric field (3.64 V/cm), temperature (87.30 °C) and pressure (0.3 bar), respectively, compared to conventional heating. The results aligned with those of Ruiz et al. [49] who studied the degree of degradation of ascorbic acid in apple puree at 50–90 °C and different concentrations of oxygen.

### 3.4. Effect of OH-VC on Lycopene Content

There was no significant deterioration in the lycopene content among all treatments. This finding was consistent with Hassen et al. [6] who determined the amount of lycopene in the semi-concentrated tomato paste at two temperatures (80 and 90 °C). Chanforan et al. [50] also reported that ohmic heating at a temperature of 96 °C for 35 min did not result in any degradation of the lycopene content of tomato sauce. The results showed that the electric field range (2.73–3.64 V/cm) caused a significant difference (*p* < 0.05) in lycopene content. This was in agreement with Wu and Kubota [51] who observed no significant differences in lycopene content by using either high or low electric field values.

ANOVA was used to analyze the adequacy of the CCD-developed model and the significance of the related factors for lycopene content (Table 4). The regression model for lycopene content was significant (*p* < 0.05) differently from the lack of fit that was not significant (*p* > 0.05), indicating that the model was adequate for predicting both values. The interaction between electric field and temperature was not significant for lycopene content (*p* > 0.05), while the interaction between electric field and pressure was significant (*p* < 0.05).

Response surface plots were generated for lycopene content (Figure S2). An increase in the electric field strength and temperature did not show a significant effect (*p* < 0.05) on the lycopene content. However, increasing the pressure with the electric field strength resulted in a significant effect (*p* < 0.05) compared to the effect of temperature and pressure. The optimized results of lycopene content were 33.93 ± 1.435 mg/kg using ohmic heating at 3.64 V/cm, 87.3 °C and 0.3 bar (Table 5) compared to conventional heating 28.51 ± 1.173 mg/kg. The results were not in agreement with Achir et al. [52] who reported that there were no significant differences between ohmic heating and conventional heating with regard to the content of carotenoids, including lycopene, for both grapefruit juice and blood oranges.

### 3.5. Influence of OH-VC on HMF

The amount of hydroxymethylfurfural ranged between 4.195 and 5.854 ppm (Table 3). This variation in the amount of hydroxymethylfurfural may be due to the treatment time and formation of the Maillard reaction. In addition, the variation could be due to the difference in the pressure values. These results were in agreement with the findings of Sabanc et al. [53] who studied the effect of ohmic heating under vacuum conditions on the hydroxymethylfurfural content of raw pomegranate juice. The results showed that the lowest content of HMF was at the highest voltage gradient, while the highest amount of HMF was at the lowest voltage.

Figure S3 illustrates the three-dimensional plot of the response surface of hydroxymethylfurfural value as a function of the electric field, temperature, and pressure. The results in Table 5 show the content of hydroxymethylfurfural under optimal conditions for ohmic heating at (3.64 V/cm, 87.30 °C, 0.3 bar) for the electric field strength, temperature and pressure, respectively. In contrast, conventional heating was at 87–90 °C. Statistical analysis showed a significant difference (*p* < 0.05) between the two treatments, as the values of HMF concentration in ohmic heating, were significantly less than those in conventional heating at 1.055 ± 0.05232 and 3.4935 ± 0.0770 ppm, respectively. Moreover, the results agreed with Pires et al. [54] who used the ohmic heating at 8–24 V/cm of electric field and 72–75 °C of temperature for 15 sec. The amount of HMF in milk using conventional heating was significantly (*p* < 0.05) higher than the amount from ohmic heating.

### 3.6. Influence of OH-VC on PME

The result of ohmic heating is shown in Table 3. The amount of pectin methyl at 1.82–3.64 V/cm of electric fields, 70–90 °C of temperatures, and 0.7–0.3 bar of pressure was very low among all treatments. The amount of PME ranged between 1.18 × 10^−4^ and 5.91 × 10^−3^ units/mL. Abedelmaksoud et al. [55] indicated the possibility of using ohmic heating as efficient heat treatment in inhibiting PME enzyme by using moderate temperatures. For example, treated mango juice using ohmic heating resulted in inhibition of pectin methylesterase by about 96%. Hernández and Cano [56] mentioned that the efficacy of inhibition of PME enzyme activity in tomato puree was high at low pressure and moderate temperatures. The results agreed with Hsu [57] who found that PME was completely inhibited in the thermally treated tomato juice.

The summarized ANOVA findings of the significant regression model are described in Table 4 for PME samples with an R^2^ of 927, suggesting that this model was an appropriate fit. Figure S4 shows the three-dimensional diagram of the response surface for the electric field, temperature, and pressure on the PME. The interaction between electric field and temperature had a significant effect (*p* < 0.05) on the enzyme. In addition, the interaction between electric field and pressure had a significant effect (*p* < 0.05), while the interaction between temperature and pressure was not significant (*p* > 0.05). Statistical analysis revealed that ohmic heating at optimum conditions of the electric field, temperature, and pressure (3.64 V/cm, 87.30 °C, 0.3 bar) was significantly superior (*p* < 0.05) compared to the conventional treatment (Table 5). The enzyme values were (0.1135 × 10^−3^ ± 0.2121 × 10^−6^ and 15 ×10^−3^ ± 0.848 × 10^−6^ unit/mL in tomato paste samples for both ohmic and conventional heating, respectively. This finding was different from that reported by Makroo et al. [58]. The inhibition of PME in tomato juice treated by ohmic heating was similar to that of conventional heating at 90 °C for a period of 5 min. However, conventional heating can reduce the activity of PME but causes a loss of nutritional value and results in the production of unwanted flavors in the product, especially when using temperatures higher than 80 °C [59]. The results were identical to those reported by Demirdöven and Baysal [60] who treated orange juice using ohmic heating at (44–42 V/cm) of electric field and (69, 70 °C) of temperatures. The ohmic heating inhibited PME by about 96%, while in conventional heating the inhibition rate was 88.3%.

### 3.7. Effects of OH-VC on the Microbial Quality

There was no growth of coliform bacteria, yeasts, and molds for both ohmic heating and conventional heating among all treatments. In contrast, the total count of bacteria varied from 0 to 2.47 log cfu/mL, and the highest bacterial growth was at 1.82 V/cm of the electric field, 70 °C, and 0.7 bar. The results also indicated that an increase in the electric field led to the inhibition of bacterial growth at each of the electric fields (2.73 and 3.64 V/cm). These results agreed with Lee et al. [61] who studied the effect of ohmic heating on *Listeria monocytogene*, *Typhimurium Salmonella,* and *Escherichia coli* O157:H7 in tomato juice with an electric field range between (25–40 V/cm) and temperature (76 °C) for (30–300) s. A decrease in the growth of microorganisms from log5 cfu/mL to log 1 cfu/mL was found. Moreover, the temperature had a clear effect on the bacterial inhibition at (80 and 90 °C), as the percentage of inhibition increased with the increase in temperature.

Table 5 shows the microbial growth of tomato paste produced under optimum conditions of ohmic heating at 3.64 V/cm of the electric field, 87.30 °C, and 0.3 bar compared to conventional heating at (87–90 °C). There was no microbial growth for either ohmic heating or conventional heating. These results agreed with those reported by Leizerson and Shimoni [62] who studied the effect of ohmic heating and conventional heating on microbial growth in orange juice. Their findings recommended the use of heat treatment to inhibit microorganisms regardless of the type of treatment. However, Lee et al. [63] indicated that conventional heating at temperatures of 50 and 60 °C did not lead to the inhibition of pathogenic bacteria, while ohmic heating inhibited microorganisms at 50, 55, and 60 °C when applied to foods with low acidity.

### 3.8. Effect of OH-VC on the Electrical Conductivity

Table S1 shows the electrical conductivity values for (20) treatments of tomato paste produced by ohmic heating with an electric field (1.82, 2.73, 3.64 V/cm), temperature (70, 80, 90 °C), and pressure (0.7, 0.5, 0.3 bar). The values of electrical conductivity ranged between 7.74051–17.8275 S/m. This change in electrical conductivity was due to the change in current, as the latter directly affects electrical conductivity values. Statistical analysis showed that there were no significant differences (*p* < 0.05) among all treatments of tomato paste regarding the electrical conductivity. The model was not significant (*p* > 0.05) as well as the lack of fit (*p* > 0.05), suggesting how that the electric field, temperature, and pressure had no significant effect on the electrical conductivity of tomato paste. According to the statistical analysis, the model was not adequate to predict productivity. The value of the coefficient of determination (R^2^) was 0.47.

Figure S5 shows the response surface for electrical conductivity. The results revealed a variation in electrical conductivity under different conditions, which was consistent with Icier et al. [64] who observed that the value of electrical conductivity increased using the ohmic heating when the temperature raised. On the other hand, the value of electrical conductivity decreased with the increasing concentration of soluble substances in fruit juices (apple, orange, carrot, and strawberry juice). These results were in agreement with [48] who studied the effect of ohmic heating on electrical conductivity in papaya pulp. The findings revealed an increase in electrical conductivity values at 20 V/cm until a temperature of 50 °C was reached. Icier et al. [64] studied the effect of ohmic heating on pomegranate juice in a vacuum. The results found a decrease in the evaporation time with an increase in the electric field. It was concluded that the highest value of electrical conductivity was obtained when a certain concentration of total solids was reached [65,66].

### 3.9. Energy Efficiency of OH-VC Combination Heating System

As can be seen in Table S1, the energy efficiency generally decreased with the increase in the electric field. For example, when the electric field was 1.82, 2.73, and 3.64 v/cm regardless of the temperature and pressure, the energy efficiency reached 64.26%, 45.41%, and 18.44%, respectively. This may be attributed to an increase in input power. ANOVA was used to analyze the adequacy of the CCD-developed model and the significance of the related factors for energy efficiency. Table S2 shows that the model has a significant effect (*p* < 0.05) on the energy efficiency, unlike the lack of fit which was not significant (*p* > 0.05). According to the statistical analysis, the model was adequate for predicting productivity. The value of the coefficient of determination (R^2^) was 0.57.

Three-dimensional plots of surface responses were generated by altering two variables within the experimental range and keeping the third variable constant at the central level (Figure S6). The results revealed that the increasing electric field and temperature led to an increase in energy efficiency, with a maximum value of 64%. The optimized result of energy efficiency was 68.17% using an OH-VC combination heating system at 3.64 V/cm of the electric field, 87.30 °C of temperature, and 0.3 bar of pressure (Table S3). There were statistically significant variations between 68.17% of the OH-VC combination heating system samples and conventional heating (49.33).

### 3.10. Specific Energy Consumption

Table S1 shows the values of specific energy consumption of tomato paste by ohmic heating for twenty treatments using an electric field ranging from (1.82–3.64 V/cm) temperature (70–90 °C) and pressure (0.7–0.3 bar). The lowest specific energy consumption was 1554.79 kJ/kg at 1.82 V/cm, 90 °C, 0.7 bar, while the highest was 17,479.9 kJ/kg at 3.64 V/cm, 70 °C, 0.3 bar. The highest value for energy consumption was at the highest electric field (3.64 V/cm). Table S2 illustrates that the model had a significant effect (*p* < 0.05) on the specific energy consumption. The value of the coefficient of determination (R^2^) was 0.928. Figure S7 shows the 3D plot of the response surface of specific energy consumption as a function of the electric field, temperature, and pressure. Figure S7A shows the effect of the interaction between temperature and the electric field at a fixed pressure of 0.5 bar. Figure S7B shows the pressure and electric field with the temperature fixed at the midpoint of 80 °C. As shown in Figure S7C, with the electric field set at 2.73 V/cm, there was increased specific energy consumption extraction at higher temperatures and pressure. The optimized result of specific energy consumption was 6701.750 ± 908.5 kJ/kg using ohmic heating at 3.64 V/cm of the electric field, 87.30 °C, and 0.3 bars of pressure (Table S3). There were statistically significant variations between the ohmic heating samples and conventional heating. The ohmic heating system saved a substantial amount of energy about 55% compared to conventional heating [67].

### 3.11. Productivity

Table S1 shows the productivity results of twenty treatments for the manufacture of tomato paste by ohmic heating. The two treatments had the highest productivity of 0.558571, 0.542759 kg/h under the conditions of the electric field, temperature, and pressure, which were 2.73 V/cm, 90 °C, 0.5 bar, and 2.73 V/cm, 80 °C, 0.3 bar respectively. Meanwhile, the lowest productivity was for 0.08156 kg/h at 1.82 V/cm, 70 °C, 0.7 bar due to the increased time required for paste concentration, slow heating, and less evaporation. The results in Table S2 indicate that the mathematical model and the lack of fit had a significant effect (*p* < 0.05) on productivity The highest productivity was 0.2763 ± 0.098 kg/h at an electric field of 87.30 °C, 3.64 V/cm and a pressure of 0.3 bar, compared to the productivity under conventional heating which required a long time to give 0.089647 ± 0.01 kg/h (Table 6). Figure S8 illustrates the three-dimensional plot of the response surface of the productivity value as a function of the electric field, temperature, and pressure. The results indicated that an increase in the temperature and the electric field led to an increase in productivity because of reducing the required time for processing tomato paste.

### 3.12. Sensory Evaluation

The results of the sensory evaluation are shown in Table 7. Taste panelists gave scores to the OH-VC-treated samples, which were 8.6 ± 0.632, 8.33 ± 0.723, 8.13 ± 0.743, 8.13 ± 0.990 and 8.13 ± 0.854 under optimized conditions (electric field of 3.64 V/cm, a temperature of 87.30 °C and a pressure of 0.3 bar) for color, texture, taste, appearance and overall acceptability, respectively. There was a significant difference (*p* < 0.05) between the OH-VC heating treated samples and conventional heating with regard to color, appearance, texture and overall acceptability. This result was in agreement with Makroo et al. [58] who found that ohmic heating was more effective in retaining a bright color and higher viscosity and consistency compared to conventional heating. In contrast, the results of the statistical analysis showed that there was no significant difference (*p* > 0.05) between the OH-VC treated samples and the conventional heating sample with respect to taste. These results were not in agreement with Tumpanuvatr and Jittanit [68] who indicated that the sensory characteristics of juice samples treated with ohmic and conventional heating were generally similar.

## 4. Conclusions

A batch OH-VC combination heating system was developed and used for producing tomato paste at temperatures of 70, 80, and 90 °C and pressure of 0.3, 0.5, and 0.7 bar and electric field of 1.82, 2.73, and 3.64 V/cm using a central composite design. The results attained indicated that ohmic heating had no effect on the degradation of ascorbic acid and lycopene contents, compared with conventional heating. The OH-VC combination yielded a minor effect on HMF content due to less exposure to heating temperature and rapid evaporation compared to conventional heating. Sensory evaluation results highlighted that OH-VC combination heating was superior to conventional heating for color, texture, appearance, and overall acceptability. The same effect was observed for specific energy consumption, energy efficiency, and productivity. In conclusion, the developed OH–VC combination heating has a great potential for utilization in the processing of food products by helping to maintain their quality and nutritional value.

## Figures and Tables

**Table 1:** Settings of variables for the experiment design.

Symbols	Independent Variables	−1	0	1
**X_1_**	Temperature (°C)	70	80	90
**X_2_**	Pressure (bar)	0.3	0.5	0.7
**X_3_**	Electrical field (V/cm)	1.82	2.73	3.64

**Table 2:** Independent variables of the study.

Run	Temperature (°C)	Pressure (Bar)	Electrical Conductivity (V/cm)
1	80	0.50	1.82
2	70	0.30	3.64
3	70	0.50	3.64
4	70	0.70	3.64
5	80	0.50	2.73
6	80	0.50	2.73
7	80	0.50	2.73
8	70	0.50	3.64
9	80	0.70	2.73
10	90	0.50	2.73
11	90	0.70	1.82
12	80	0.30	3.64
13	80	0.50	1.82
14	80	0.30	2.73
15	80	0.50	2.73
16	90	0.70	1.82
17	90	0.30	2.73
18	70	0.30	2.73
19	90	0.50	2.73
20	80	0.70	1.82

**Table 3:** Experiment design and responses of pH, titratable acidity %, TSS brix, density kg/m^3^, ascorbic acid AA mg/100 g, lycopene mg/kg, HMF ppm, and PME unit/mL.

Independent Variables				pH	Titratable Acidity	TSS	Density	AA	Lycopene	HMF	PME
RUN	Electric Field (V/cm)	Temperature	Pressure (Bar)								
		(°C)									
1	1.82	70	0.7	4.2	0.33	26	1074.07	61.6	27.53	5.854	0.0000541
2	3.64	90	0.3	4.4	0.34	29	1075.26	48.42	27.53	1.561	0.0000581
3	3.64	90	0.7	4.2	0.39	29	1075.26	22	24.79	4.972	0.0000.225
4	3.64	70	0.3	4.1	0.34	28	1079.79	44	26.26	4.195	0.0000751
5	2.73	70	0.5	4.1	0.38	27	1074.07	66.35	24.78	5.718	0.0000344
6	2.73	80	0.5	4.2	0.33	27	1068.93	44.2	26.12	1.886	0.0000585
7	2.73	80	0.5	4.2	0.32	27	1068.93	43.98	26.25	1.861	0.0000571
8	3.64	70	0.7	4.3	0.39	28	1079.79	27.84	25.74	3.851	0.0000545
9	2.73	80	0.5	4.2	0.36	27	1068.93	44.34	26.91	1.561	0.0000408
10	2.73	90	0.3	4.2	0.37	29	1075.26	57.61	27.97	1.587	0.0000118
11	1.82	80	0.5	4.2	0.35	27	1068.93	26.66	25.89	3.48	0.0000456
12	3.64	80	0.5	4.3	0.36	28	1074.66	52.31	27.33	4.371	0.0000445
13	1.82	90	0.7	4.1	0.46	26	1058.08	22.03	24.21	3.731	0.000636
15	2.73	80	0.7	4.2	0.41	26	1058.08	40.51	25.67	1.783	0.0000362
16	2.73	80	0.3	4.1	0.36	27	1068.93	66.17	26.15	1.82	0.0000511
17	1.82	70	0.3	4.2	0.33	28	1074.66	67.6	26.77	1.725	0.0000582
18	2.73	80	0.5	4.2	0.33	26	1068.34	43.88	28.18	3.48	0.000591
19	2.73	80	0.5	4.2	0.39	28	1074.66	44.51	26.85	1.587	0.0000525
20	2.73	80	0.5	4.2	0.38	27	1068.93	43.95	26.23	1.908	0.0000543

**Table 4:** Regression coefficients, R^2^, and *p* values of the model for eight dependent variables for OH-VC combination heating samples.

Regression Coefficient	pH	T.A	Density	A.A	Lycopene	HMF	PME
b_0_	4.19106	0.978	658.3262	201.0785	269.6063	94.7595	0.017990129
b_1_	0.050487	0.1848	46.91637	−57.1125	−142.694	−10.2135	−0.004534519
b_2_	0.050112	−0.02523	5.641375	−1.46955	−4.91786	−1.96993	−0.000283933
b_1_b_2_	−0.04996	0.11	1510.327	146.165	−3.59318	12.27199	−0.018056761
b_1_^2^	0.037312	−0.00234	−0.78719	0.661925	2.633469	0.034416	5.23476 × 10^−5^
b_2_^2^	0.012738	0.00021	−118.078	−0.6875	17.30437	−0.99756	0.005480062
b_1_b_2_^2^	−0.06261	---	−20.0375	−2.49	−0.5	0.220313	0.0002211
b_1_^2^ b_2_	0.072873	-	3.030672		10.85322	1.47565	0.000114884
R^2^	0.982955	0.666402	0.873249	0.676866	0.945965	0.697817	0.927586
*p*-value of the model	0.0003	0.0049	0.0056	0.0107	0.0087	0.0792	0.0006
*p*-value of lack of fit		0.8048	0.7833	0.0001	0.0992	0.4158	0.0001

**Table 5:** Mean of dependent variables OH-VC combination heating under optimized conditions of 3.64 V/cm, 87.30 °C, and 0.3 bars compared with conventional heating.

Dependent Variable	OH-VC	Conventional Heating
AA (mg/100 g)	67.76 ± 1.244 ^a^	50.16 ± 1.244 ^b^
Lycopene (mg/kg)	33.93 ± 1.435 ^a^	28.51 ± 1.173
HMF (ppm)	1.055 ^a^ ± 0.052	3.493 ± 0.77 ^b^
PME (unit/mL)	0.1135 × 10^−3^ ± 0.2121 × 10^6^	0.15 ×10^−3^± 0.848528 × 10^−6^ ^b^
pH	4.2 ^a^	4.3 ^a^
Titratable acidity (%)	0.42 ± 0.42 ^a^	0.35 ± 0.0141 ^b^
TSS (brix)	27.5 ± 0.707 ^a^	28 ± 1.414 ^a^
Total plate count	0	0
*E. Coli*	0	0
Yeast and mold	0	0

^a–b^ Means with same superscripts across a row are not significantly different.

**Table 6:** Mean of dependent variables for ohmic-vacuum (OH-VC) combination heating under optimized conditions of 3.64 V/cm, 87.30 °,C and 0.3 bar compared with conventional heating.

Dependent Variable	OH-VC	Conventional Heating
Electrical Conductivity (V/cm)	13.08 ± 2.01 ^a^	_
Efficiency (%)	36.87 ± 2.985 ^a^	1.3207 ± 0.12 ^b^
SEC (kJ/kg)	6701.750 ± 908.5 ^a^	31,233.33 ± 805.65 ^b^
Productivity kg/h	0.2763 ± 0.098 ^a^	0.089647 ± 0.01 ^b^

^a–b^ Means with same superscripts across a row are not significantly different.

**Table 7:** Sensory evaluation scores of the ohmic heating treated samples under optimum conditions at an electric field strength of 3.64 V/cm, a temperature of 87.30 °C, and a pressure of 0.3 bar, and the conventional heating.

Sample	Color	Texture	Taste	Appearance	Overall Acceptability
OH-VC	8.6 ± 0.632 ^a^	8.33 ± 0.723 ^a^	8.13 ± 0.743 ^a^	8.13 ± 0.990 ^a^	8.13 ± 0.854 ^a^
Conventional heating	6.63 ± 0.972 ^b^	7.3 ± 1.130 ^b^	8.03 ± 0.718 ^a^	7.8 ± 0.797 ^a^	7.36 ± 0.854 ^b^

^a–b^ Means with same superscripts across a row are not significantly different.

## Data Availability

All data are reported in this manuscript.

## Supplementary Materials

The following are available online at https://www.mdpi.com/article/10.3390/foods10122920/s1, Figure S1: Response surface model plot showing the effects of independent variables on the ascorbic acid (mg/100 mL sample): panel A temperature and electric field; panel B pressure and electric field; and panel temperature and pressure, Figure S2: Response surface model plot showing the effects of independent variables on the lycopene content (mg/kg): panel A temperature and electric field; panel B pressure and electric field; and panel temperature and pressure, Figure S3: Response surface model plot showing the effects of independent variables on HMF (ppm): panel A temperature and electric field; panel B pressure and electric field; and panel temperature and pressure., Figure S4: Response surface model plot showing the effects of independent variables on PME (unit/mL): panel A temperature and electric field; panel B pressure and electric field; and panel temperature and pressure, Figure S5: Response surface model plot showing the effects of independent variables on EC (S/cm): panel A temperature and electric field; panel B pressure and electric field; and panel temperature and pressure., Figure S6: Response surface model plot showing the effects of independent variables on the energy efficiency (%).,Figure S7: Response surface model plot showing the effects of independent variables on SEC (kj/kg): panel A temperature and electric field; panel B pressure and electric field; and panel temperature and pressure, Figure S8: Response surface model plot showing the effects of independent variables on the productivity (kg/h).,Table S1: Experimental design and responses of electrical conductivity (S/m), Efficiency (%), Specific energy consumption (kj/kg), and Productivity (Kg/h), Table S2: Regression coefficients, R^2^, and *p* values of the model for four dependent variables for ohmic-vacuum (OH-VC) combination heating samples.
