# Supplementary material for: Application and Effects of Ohmic-Vacuum Combination Heating on the Quality Factors of Tomato Paste
Source: Foods. 2021 Nov 25;10(12):2920. doi: 10.3390/foods10122920 (PMC8700374; doi:10.3390/foods10122920)
Supplement: Supplementary file 1 [file foods-10-02920-s001.zip › foods-1423712-supplementary.pdf]

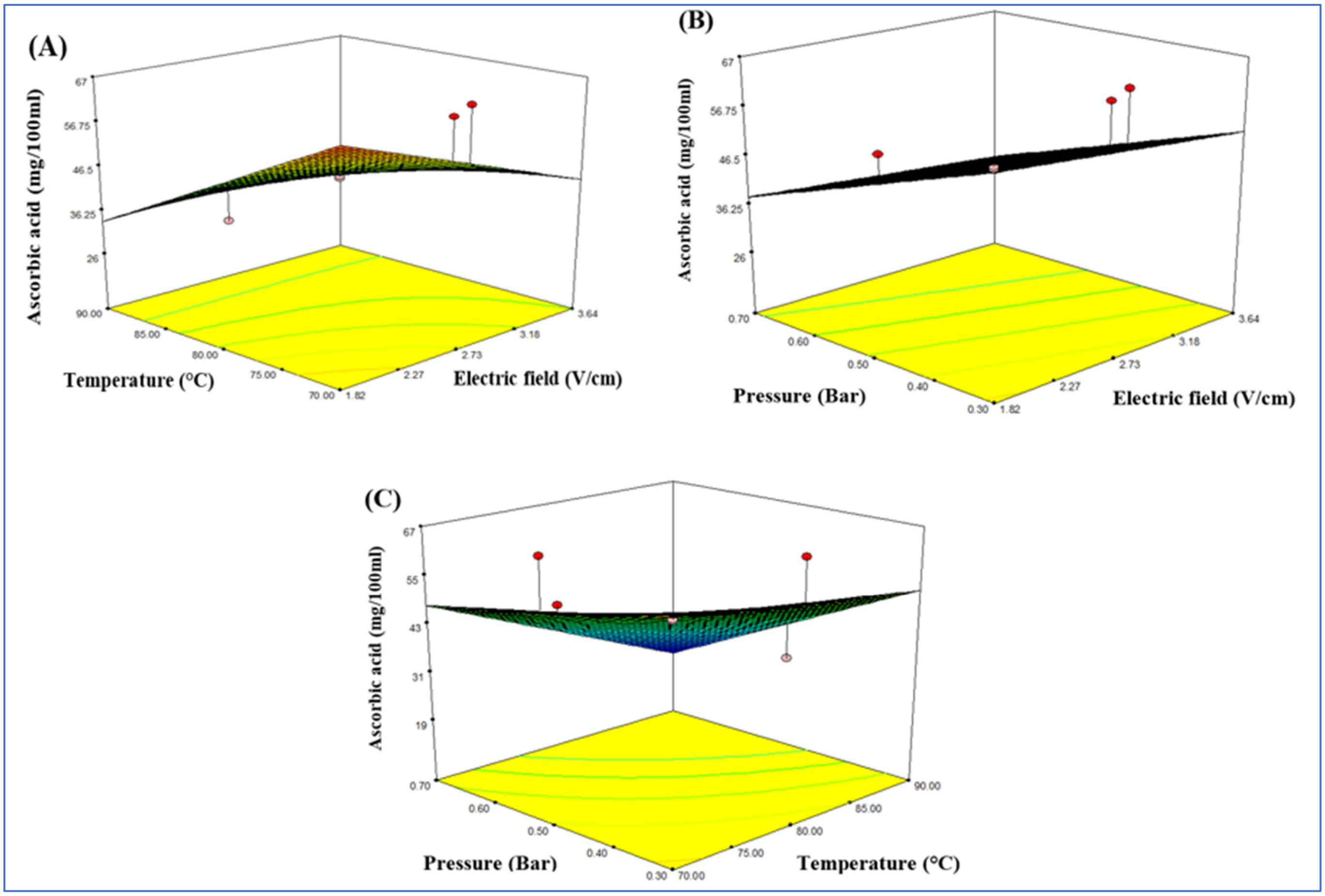

Figure S1. Response surface model plot showing the effects of independent variables on the ascorbic acid (mg/100 ml sample): panel A temperature and electric field; panel B pressure and electric field; and panel temperature and pressure.

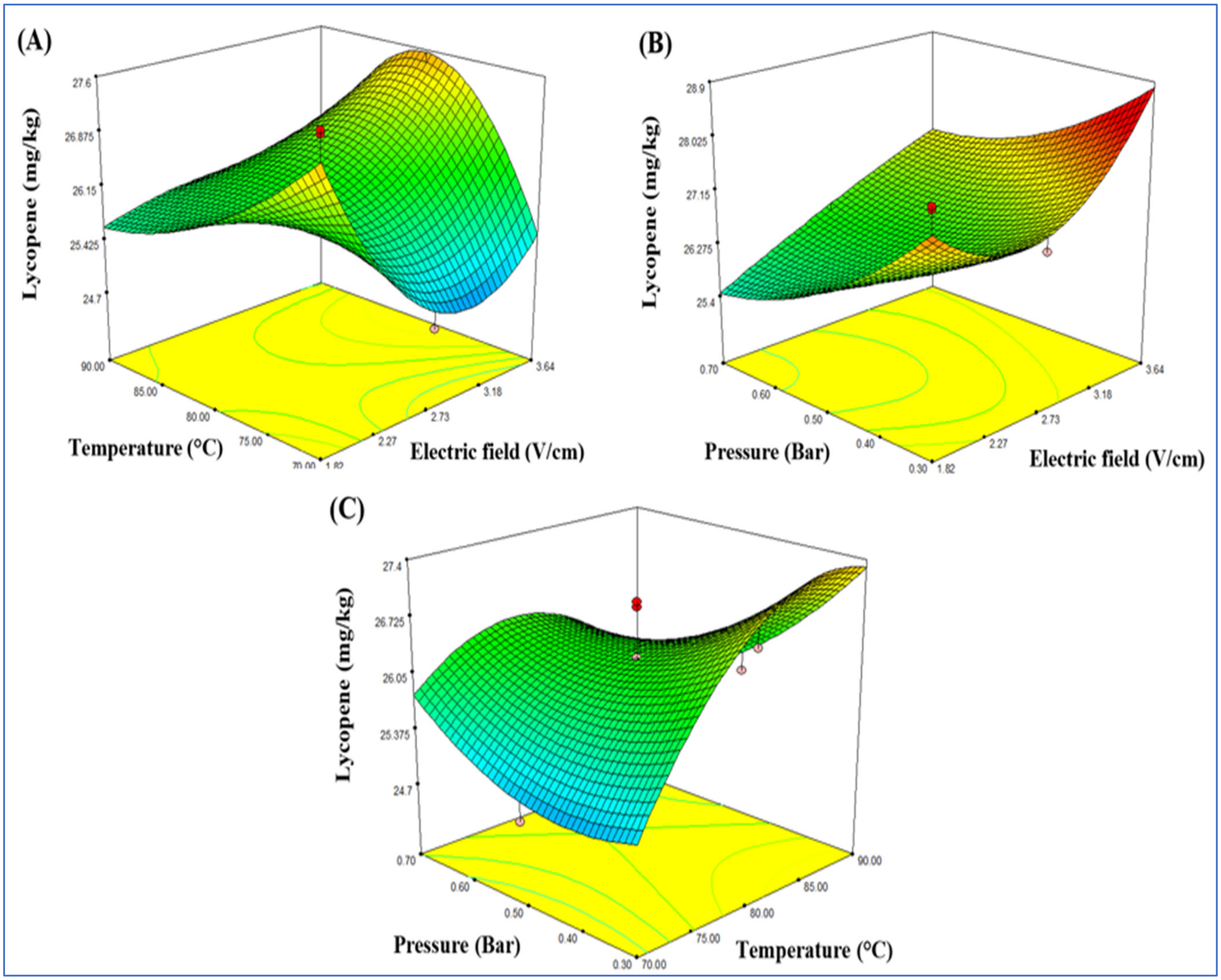

Figure S2. Response surface model plot showing the effects of independent variables on the lycopene content (mg/kg): panel A temperature and electric field; panel B pressure and electric field; and panel temperature and pressure.

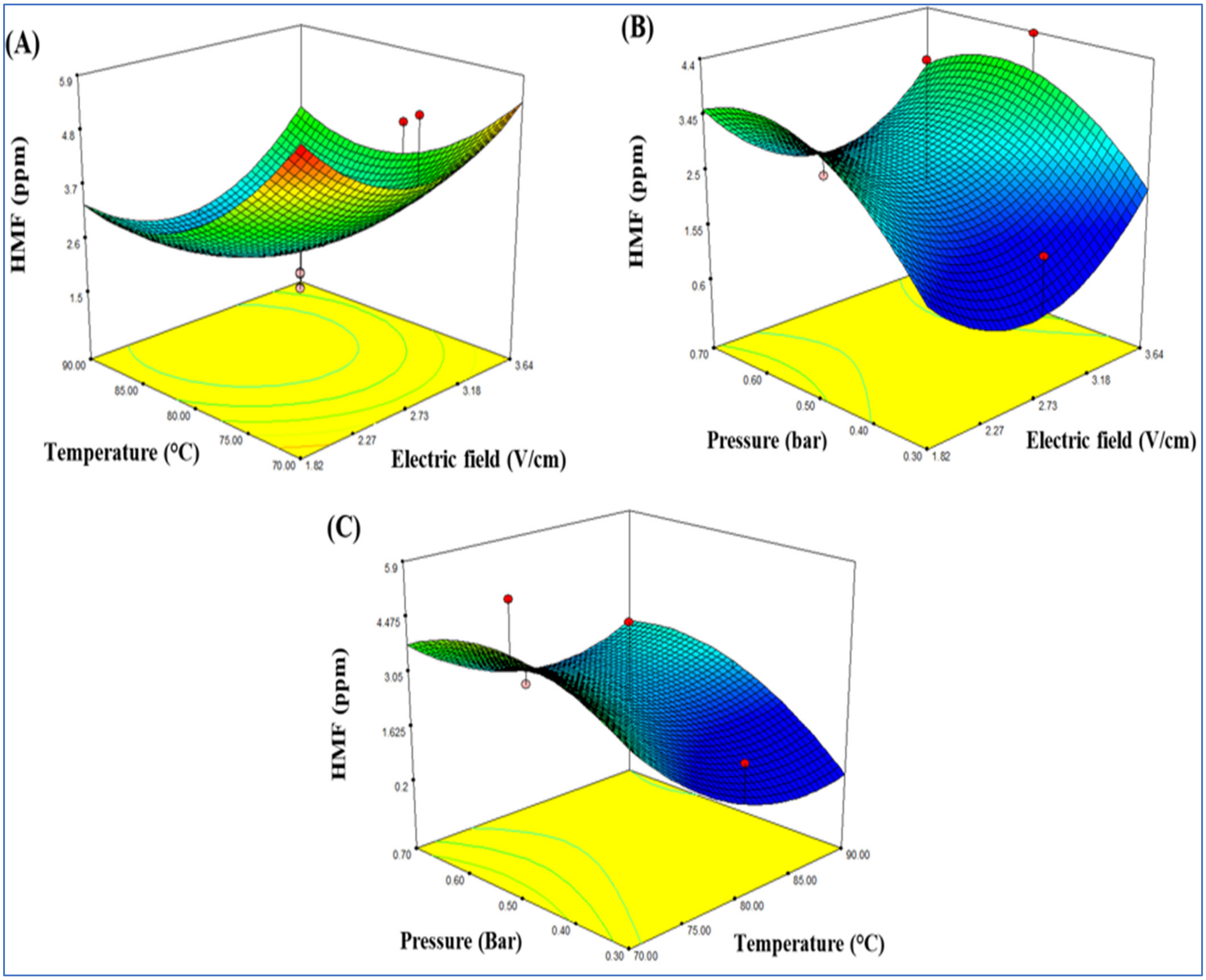

Figure S3. Response surface model plot showing the effects of independent variables on HMF (ppm): panel A temperature and electric field; panel B pressure and electric field; and panel temperature and pressure.

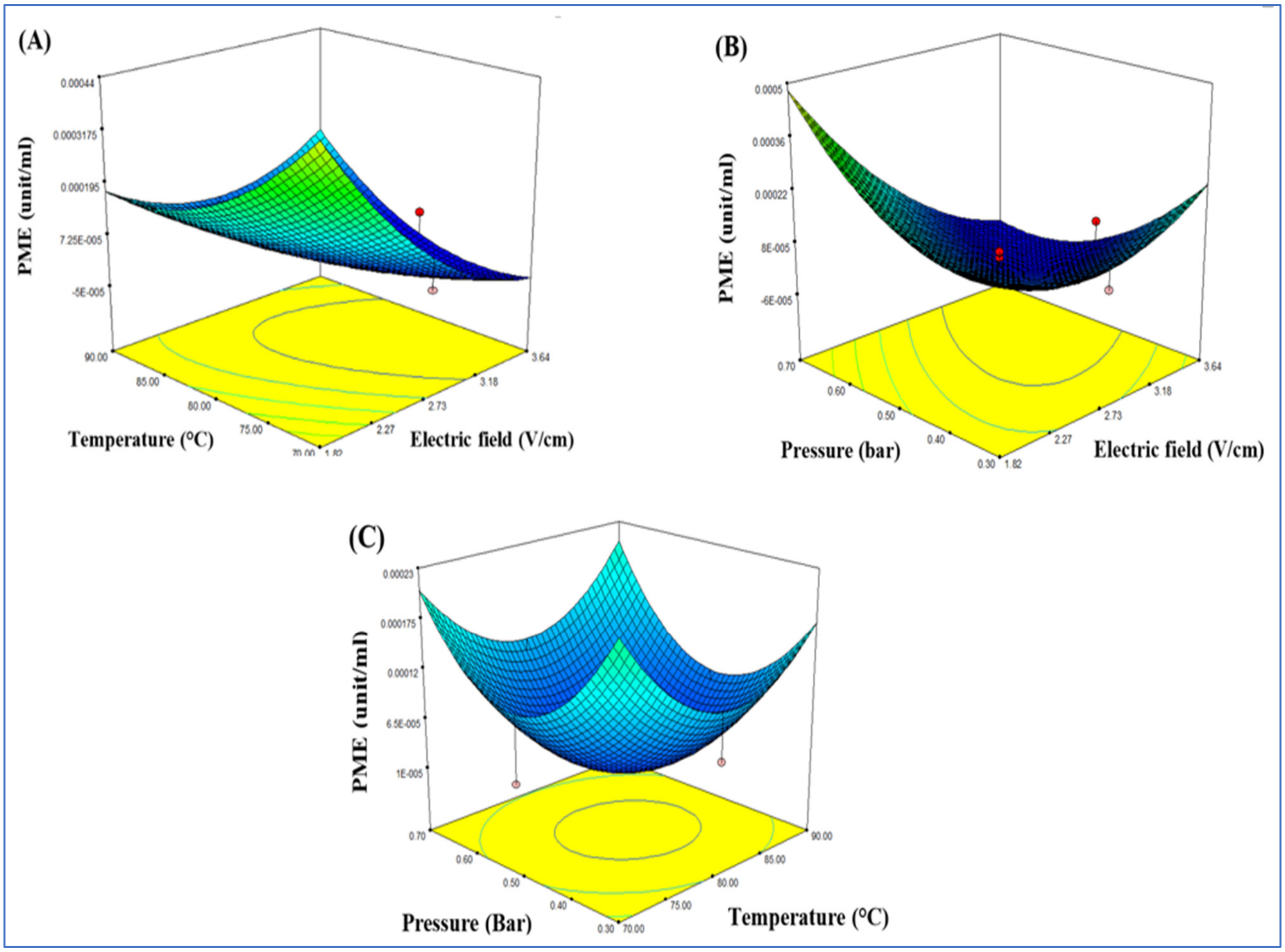

Figure S4. Response surface model plot showing the effects of independent variables on PME (unit/ml): panel A temperature and electric field; panel B pressure and electric field; and panel temperature and pressure.

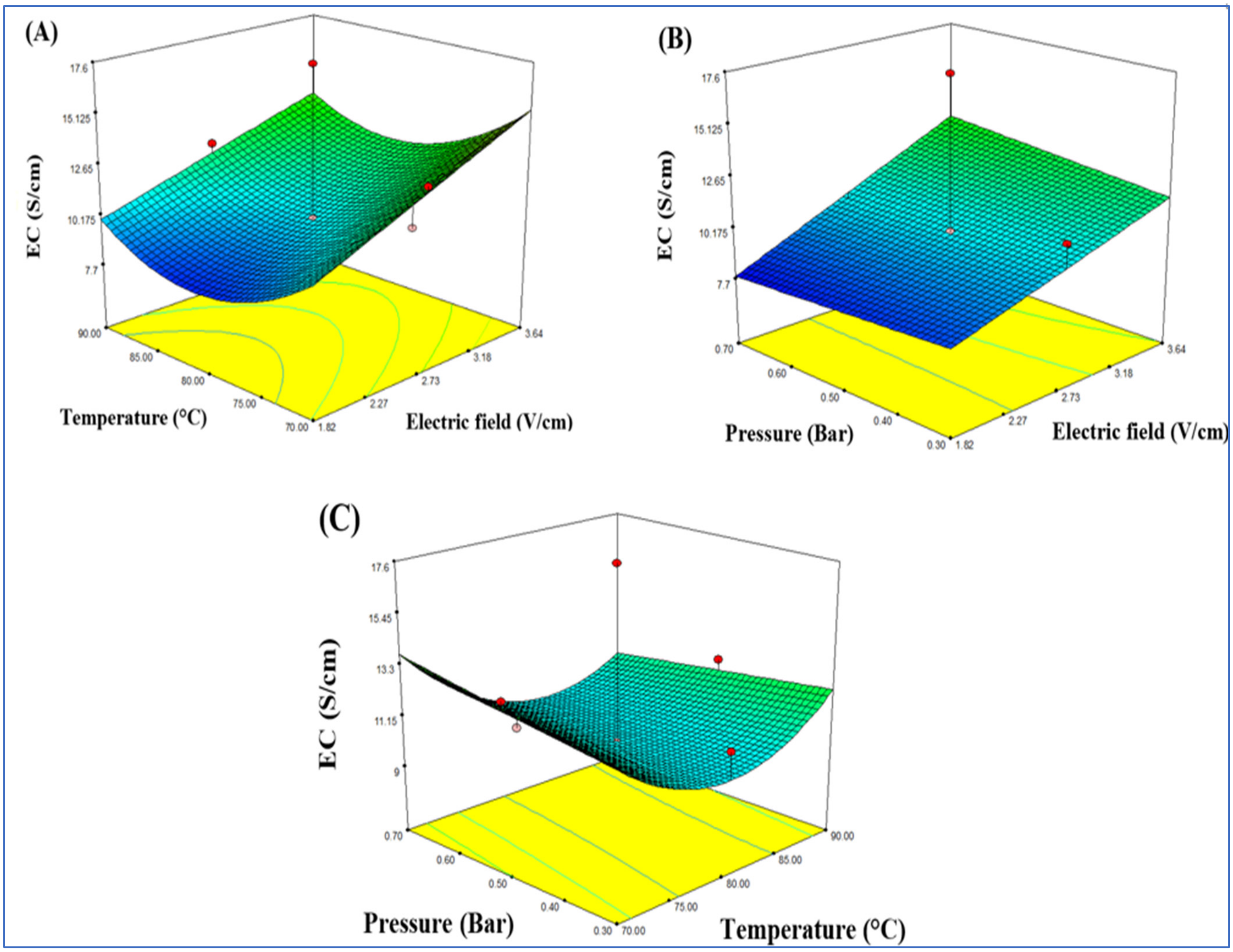

Figure S5. Response surface model plot showing the effects of independent variables on EC (S/cm): panel A temperature and electric field; panel B pressure and electric field; and panel temperature and pressure.

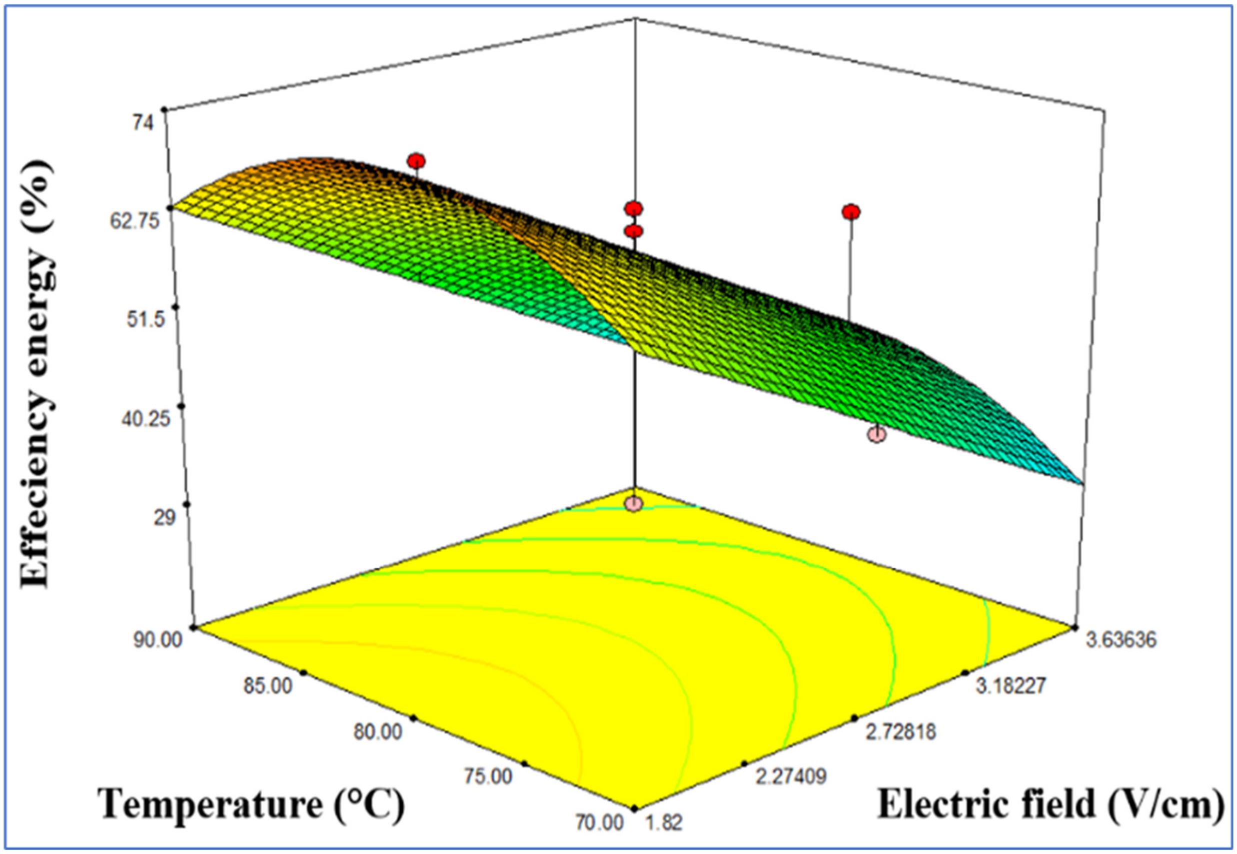

Figure S6. Response surface model plot showing the effects of independent variables on the energy efficiency (%)

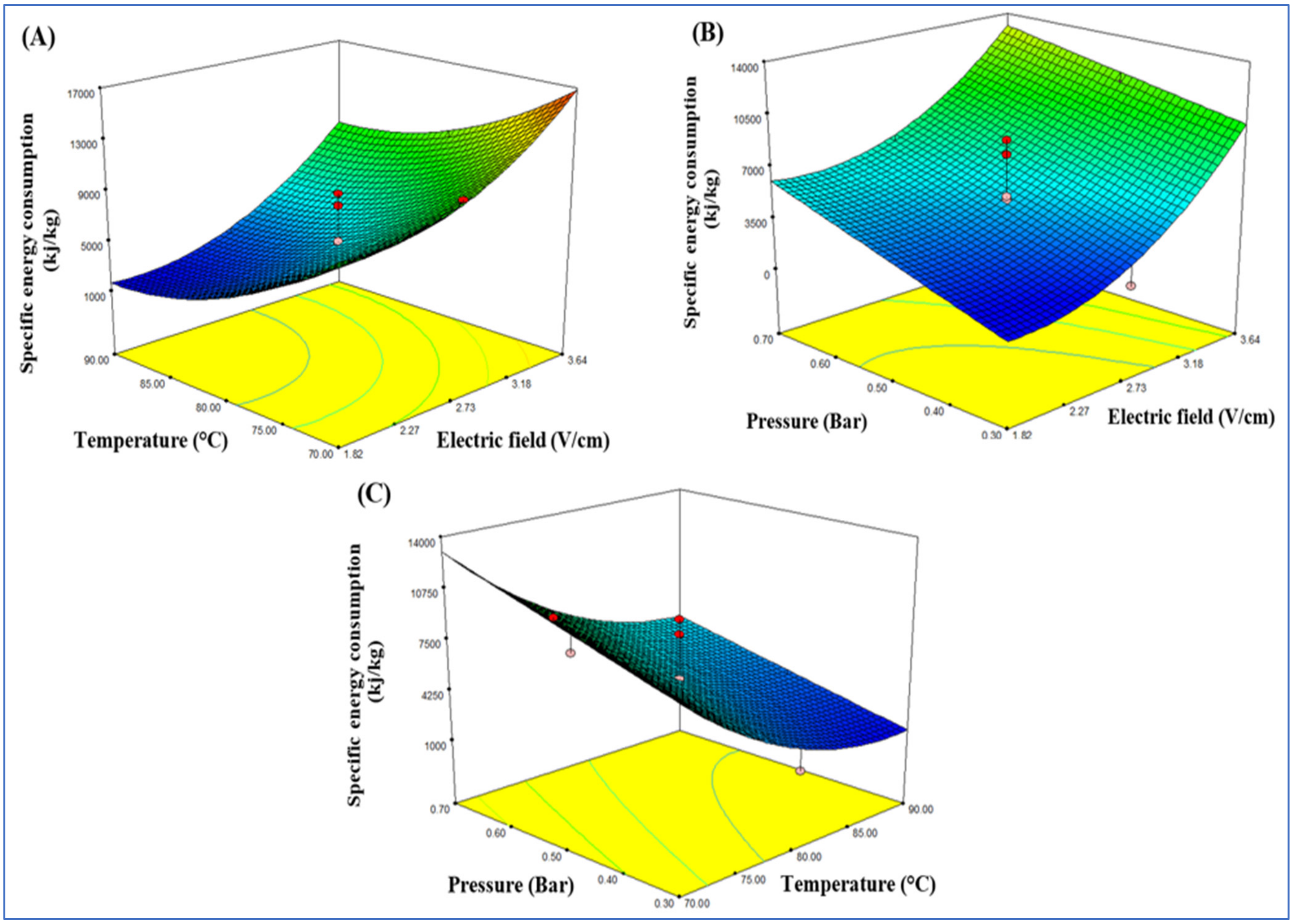

Figure S7. Response surface model plot showing the effects of independent variables on SEC (kj/kg): panel A temperature and electric field; panel B pressure and electric field; and panel temperature and pressure.

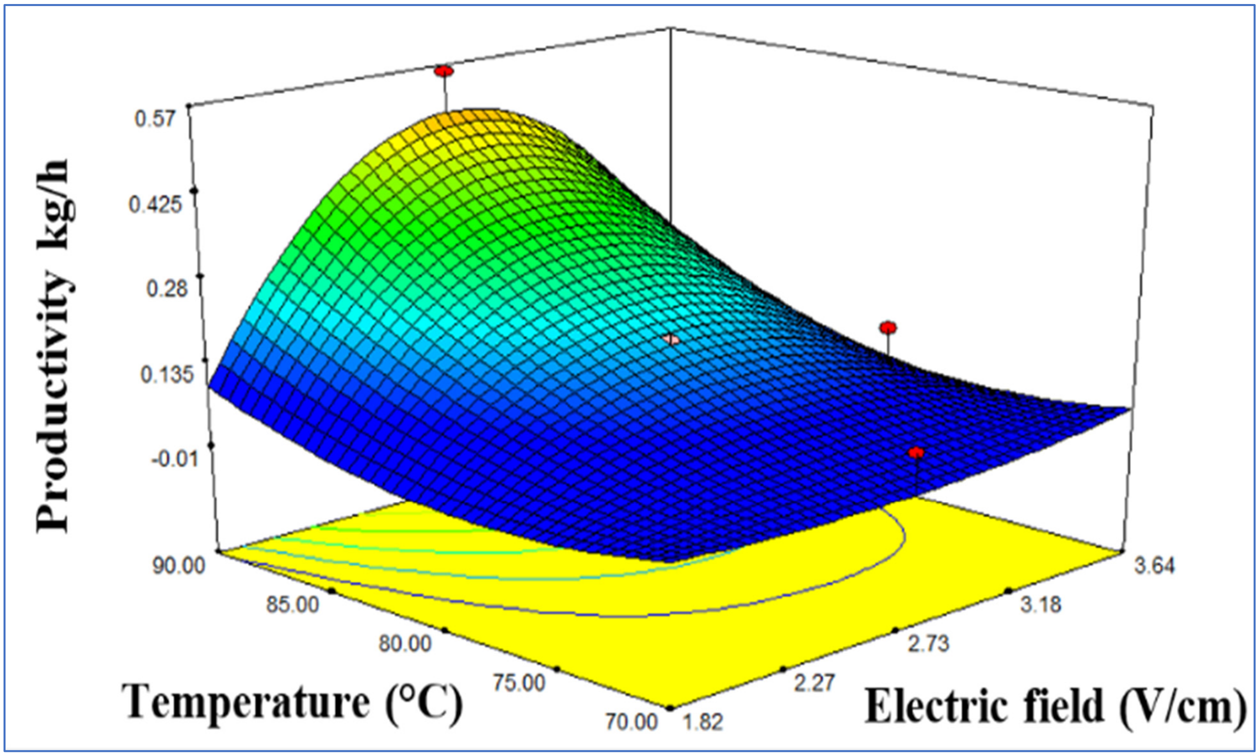

Figure S8. Response surface model plot showing the effects of independent variables on the productivity (kg/h)

Table S1. Experimental design and responses of electrical conductivity (S/m), Efficiency (%), Specific energy consumption (kj/kg), and Productivity (Kg/h)

RUN	Independent variables			Electrical conductivity	Effeciency	Specific energy consumption	Productivity
	Electic field (V/cm)	Temperature (°C)	pressure (Bar)				
1	1.82	70	0.7	10.1585	53.5567	15042.9	0.08156
2	3.64	90	0.3	14.2513	18.4441	6302.34	0.314583
3	3.64	90	0.7	13.7188	29.0105	14778.5	0.131667
4	3.64	70	0.3	13.8958	38.8024	17479.9	0.106345
5	2.73	70	0.5	13.2659	45.4181	10943.9	0.091084

6	2.73	80	0.5	17.5178	29.0546	8853.33	0.147573
7	2.73	80	0.5	9.68208	63.0713	7887.58	0.091205
8	3.64	70	0.7	17.8275	26.6385	16107.6	0.13945
9	2.73	80	0.5	10.0174	60.5941	5025.5	0.153346
10	1.82	90	0.3	9.53871	65.7436	2502.1	0.114129
11	1.82	80	0.5	8.45029	69.8637	3018.9	0.088757
12	3.64	80	0.5	7.74051	56.2944	10645.3	0.09775
13	1.82	90	0.7	8.97704	64.2679	1554.79	0.19075
14	2.73	90	0.5	12.2025	62.3614	1682.84	0.558571
15	2.73	80	0.7	9.09792	79.7121	4567.7	0.139065
16	2.73	80	0.3	11.2355	62.6228	1576.82	0.542759
17	1.82	70	0.3	9.76251	69.1906	3223.96	0.103087
18	2.73	80	0.5	9.13039	55.6602	4756.64	0.148868
19	2.73	80	0.5	9.31516	52.8869	4299.75	0.175165
20	2.73	80	0.5	9.7508	54.2315	4914.3	0.139513

Table S2. Regression coefficients, R^2, and p values of the model for four dependent variables for ohmic-vacuum (OH-VC) combination heating samples

#

Regression coefficient	EC	Energy Efficiency	Specific energy consumption	Productivity Kg/h
b_0	130.1054	-608.118	-12787.5	11.24347
b_1	3.617486	-16.8776	49814.05	-5.97621
b_2	-3.27045	17.75147	-1308.41	-0.25207
b_1b_2	20.97356	-0.11056	371357.8	6.52143
b_1^2	-0.03228	-	-753.961	0.12892
b_2^2	2.450765	-	-126971	-6.4111

**$b_1b_2^2$**	-0.33887	-	-4422.62	-7.37E-03
**b_1^2 b_2**	0.021661	-	2969.06	1.09664
**R^2**	0.471579	0.570429	0.928574	0.8238
**p-value of the model**	0.2469	0.0030	0.0001	0.0466
***p*-value of lack of fit**	0.8509	0.5812	0.4949	0.0019
